# Lump Jaw—Continued

**Published:** 1892-04

**Authors:** R. W. Hickman


### LUMP JAW—CONTINUED.

By R. W. Hickman, V. M. D.

In the Journal for March will be found criticisms entitled, "Actinomycosis and U. S. Meat Inspection," by W. L. Williams, V. S., Vice-President of the Purdue University, La Fayette, Ind., which, upon the whole, are rather a failure, and yet in concluding, the Doctor does make an attempt at truthfulness, although he tries to make me appear a prestidigitator, claiming that I take a diseased animal, decapitate it and render the remainder of the carcass sound. But I don't, because I would be as complete a failure at sleight of hand, as he is at juggling. He will remember, possibly, that those fine fat cattle offered for export, that have visible evidence of the diseases are rejected at the tagging chute and held for post mortem (*so I could see, provided I had instruments of sufficiently high power*), whether the viscera are affected, that is, whether the disease had become disseminated, (I think that is the language that I used), but if it is found that it is local, and all evidence of the disease is removed by decapitation, the remainder of the carcass is sound; but I would not for a moment have Dr. Williams think I render it so. I desire to express my thanks to Dr. Olof Schwarzkopff, and my appreciation of his excellent paper, commenced in the February number of the Journal, and concluded in the March issue, and hope he will often allow the readers of the Journal the privilege of hearing from him through its pages. And I should feel so gratified if I only knew it would be read by Dr. Williams, who in his abortive attempt at wit (page 179 of the Journal), says, "One would be led to infer that Dr. Hickman had made an extensive study of the disease" (actinomycosis); and he lays special emphasis upon the fact that "he has been furnished with one of the renowned Zeiss microscopes, with all of the latest attachments, including Dr. Norgaard and an immersion lense." I would suggest that even with all these, including the two latter, with several other possibly better known and more distinguished doctors added, it would be difficult to see the brilliancy or practical common sense in either Dr. Williams' criticisms of the testimony in the Peoria trial, or his discussion of the subject. It is possible, however, if he gives the same attention to, and makes the same effort to dissect and study the able article from the pen of Dr. Schwarzkopff, and displays a little more power of comprehension so that he can make the impression upon the minds of intelligent people that he seems desirous of, he might some day, if he lives to be old enough, become an authority. This undoubtedly appears to be his ambition. But "what fools we mortals be." I regret that Dr. Williams is obliged to inquire what is meant by the condemnation of animals on scientific principles, and am obliged to assume that the balance of the veterinary profession will be able to comprehend this, and trust that Dr. Williams will, eventually, be able to reach that point. I don't know what these people will accuse me of next. It has also been publicly announced that in my testimony at the Peoria trial I gave the size of the parasite actinomyces bovis as larger than the red blood corpucle, and that it was therefore impossible for it to pass through the small blood vessels. Now, upon the contrary, I made no comparisons of the relative sizes of the red or white blood cells and the parasite. I was asked by Judge Stevens, the able attorney for the prosecution in the direct examination: Question. What is the size of the parasite actinomyces bovis? Answer. About one three-hundredth of an inch. Question. What is the diameter or calibre of the capillary blood vessels. Answer. From one twenty-five hundredth ($\frac{1}{2500}$) to one three thousandth ($\frac{1}{3000}$) of an inch. The answers were, I believe, correctly as well as concisely given, and were not subsequently alluded to either in my direct or cross-examination, but evidently set the witness for the defense to thinking, whether they had done anything in that line previously or not. I was the first witness called, having made the request that my testimony be taken first, so that I could return to Chicago; consequently, was denied the instruction received by Dr. Williams and others, who were able to hear the balance of the testimony, and were driven through these questions asked by Judge Stevens and answered by myself, as to the relative size of the parasite as a whole and the capillary blood vessels, to procure specimens from affected animals and attempt to mount them with an idea of ascertaining upon what grounds they could overcome this evidence. It is highly improbable, though, that any of them knew much about the spores, or even of the parasite as a whole, at that time. But as I predicted in my first article in the February number of the JOURNAL OF COMPARATIVE MEDICINE AND VETERINARY ARCHIVES, the correctness of which is being already demonstrated, the noted trial known as the Peoria Lumpy Jaw case —Greenhut *vs.* Pearson—would, if no other benefit were gained than that of creating a new and intense interest in the disease, and more particularly in the nature and life history of the actinomyces bovis, be of almost inestimable value both to the stockmen and the veterinary profession. Dr. Williams further asserts that I have taken a definite stand in this matter, which is another perfectly correct statement, which stand, I am free to say, I am decidedly disposed to continue to occupy at least until future investigation shall disprove its correctness. I further assert that the stand taken by myself, if truthfully represented, is in full accord with that occupied by Dr. Casewell, State Veterinarian, whom I accompanied through the yards on Saturday, March 12, in his rounds of inspection of animals, the accumulation of the week that had been held by his assistant, Mr. McDonald, to receive, in the well-marked cases, his condemnation, and the others, in which there was simply a tumor in the jaw or neck, a post-mortem examination. In the course of our conversation, which has always been most friendly, I asked Dr. Casewell what he would do with those animals which simply showed the disease locally in the jaw or neck, that were held for post-mortem, provided there were no internal lesions. He replied emphatically and in so many words, "I pass them."

I would just like to suggest that the readers of the JOURNAL try to imagine the ludicrousness of the position, and the picture presented by Prof. Williams and others while making examinations of sections made from actinomycotic tumors, procured at the Peoria distilleries, during the progress of the trial. It would be quite safe to assume that they took the detached club forms for pores; it is particularly amusing to me that this question of the prosecuting attorney should have driven these men to a microscope as soon as they could escape from the court room, and they will admit that Judge Stevens displayed great shrewdness in asking this question, whether they ever inform us as to what they were looking for, what stains they used, and the results (honestly) of their investigations at that time. The apparent discrepancies credited to my article, my testimony and the opinion of the Honorable Secretary of Agriculture are entirely the outgrowth, either of Prof. Williams' fertile imagination or desire to misrepresent one who through force of circumstances of law was obliged to oppose him as one of the leading witnesses for the defense. Though I have no apology to offer Dr. Williams, the delicacy of my position in that noted trial will be understood, as it was an action to recover damages from the State authorities for cattle condemned and slaughtered by them.

